# Endobronchial Lipoma: An Unusual Cause of Bronchial Obstruction

**DOI:** 10.1155/2011/939808

**Published:** 2011-09-29

**Authors:** Dianbo Cao, Yutian Sun, Sirui Yang

**Affiliations:** ^1^Department of Radiology, The First Hospital of Jilin University, Jilin Province, Changchun 130021, China; ^2^Department of Pharmacy, China-Japan Union Hospital of JiLin University, Jilin Province, Changchun 130051, China; ^3^Department of Cardiovascular Center, The First Hospital of Jilin University, Jilin Province, Changchun 130021, China

## Abstract

Endobronchial lipoma is a rare benign tumor. It is difficult to differentiate benign endobronchial lipoma from their malignant counterparts, as their symptoms and complications are almost alike. Here, we describe the clinical and radiological features of EL in two cases. Multislice CT (MSCT) may play an important role in the diagnosis for EL.

## 1. Introduction

Benign tumors of tracheobronchial tree are much less common and need to be differentiated from malignant airway neoplasms, but many neoplasms are clinically and radiographically indistinguishable from malignant lesions. Among benign lesions, endobronchial lipoma is a very rare disease entity, accounting for only 0.1%–0.4% of all bronchial tumors [1, 2]. As known, lipomas are composed exclusively or nearly exclusively of mature fat. CT examination is highly specific and sensitive for the detection of fat, and identification for adipose density within the lesion convincingly narrows the differential diagnosis to endobronchial lipoma or lipomatous hamartoma. For example, homogenous fat attenuation on CT strongly suggests lipoma. Here, we present the MSCT and endoscopy imaging findings in two patients with EL, which will deepen our recognition for the diagnosis and treatment principle of EL. Based on review of literatures and clinical analysis of our cases, bronchoscopic biopsies sometimes are unsatisfactory for fat-containing lesions owing to surface inflammation and inadequate deep sample.

## 2. Clinical Summary

### 2.1. Case  1

**Figure 1:**
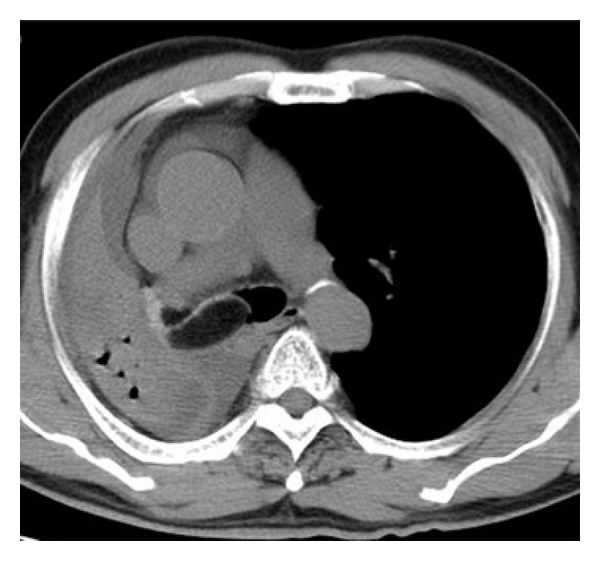
Axial CT showed hypodense mass in the right main bronchus.

**Figure 2:**
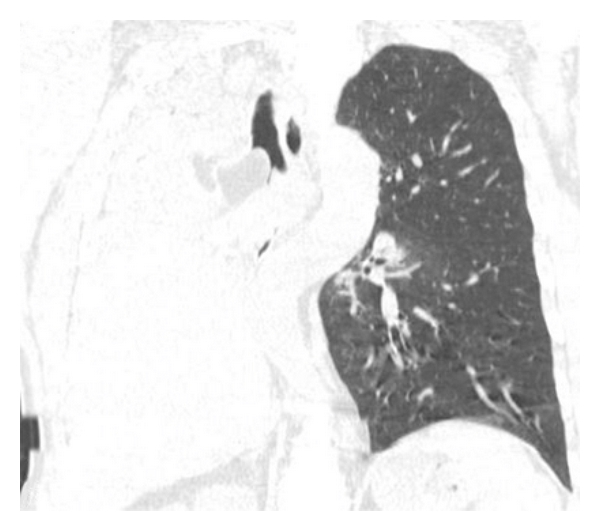
Reformatted reconstruction showed adipose mass occluding the right main bronchus and right lung atelectasis.

**Figure 3:**
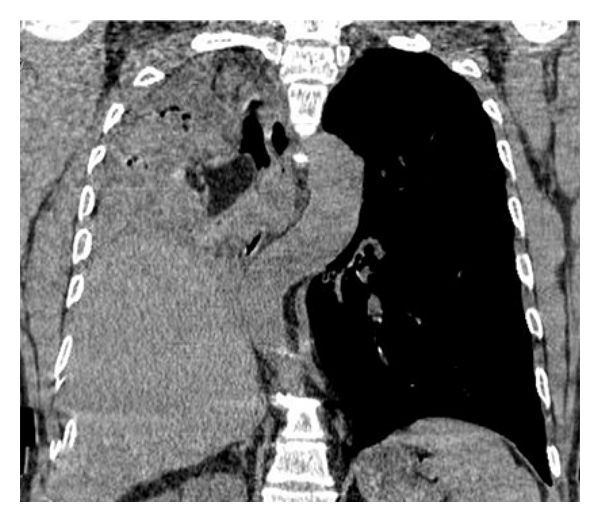
Reformatted reconstruction showed adipose mass occluding the right main bronchus and right lung atelectasis.

**Figure 4:**
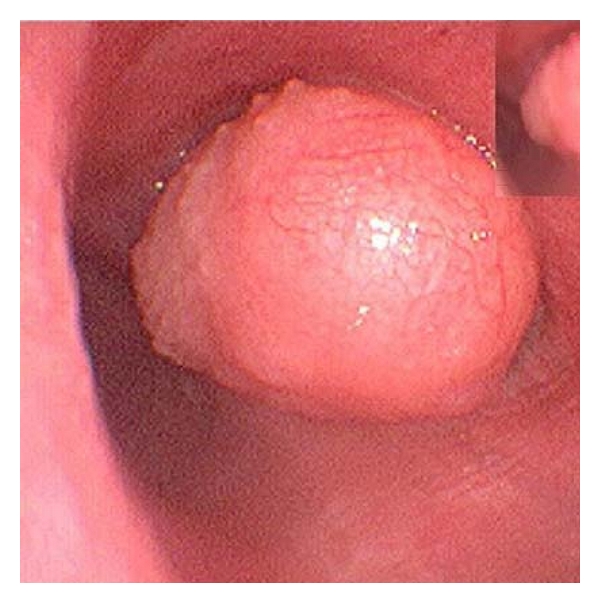
Endoscopy showed soft mass with irregular surface in the right bronchus.

A 74-year-old man was admitted to our hospital complaining of irritant cough, shortness of breath, and intermittent fever for 3 months and had also recurrent episodes of pneumonia. Physical examination of the chest revealed dullness on percussion and decreased respiratory sounds in the right lung. Chest radiography showed total atelectasis of the right lung, and MSCT scan demonstrated an endoluminal mass in the right main bronchus, right upper lobe bronchus, and bronchus intermedius. The CT value of lesion ranged from −100 to −114 Hu, and no calcification was found within the tumor (Figures 1, 2, and 3). There was also a minimal right pleural effusion. A well-circumscribed polypoid mass obstructing the right main bronchus was seen by fiberoptic bronchoscopy (Figure 4), and biopsy was performed simultaneously. Biopsy specimens showed only acute inflammatory reactions associated with squamous metaplasia. Because of indeterminate biopsy specimen and total atelectasis complicated with postobstructive pneumonia, exploratory thoracotomy was done. 

After opening the right main bronchus, an encapsulated yellow mass within the right main bronchus extending into right upper lobe and intermediate bronchus was seen, and the stalk of mass was found at the opening of right upper lobe. The mass measuring 1.5 cm × 4.5 cm was successfully excised. On microscopic examination, the tumor was made of groups of mature adipocytes. The postoperative course was uneventful, and the patient was discharged from hospital at 10th day and had been well at 24-month followup.

### 2.2. Case  2

**Figure 5:**
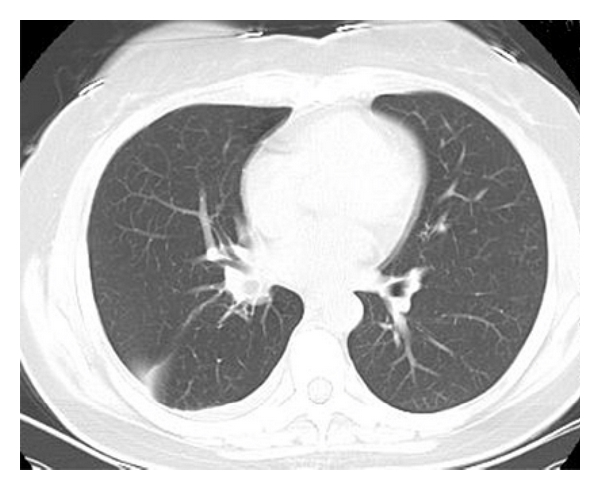
Axial CT showed the occlusion of right inferior basal branch.

**Figure 6:**
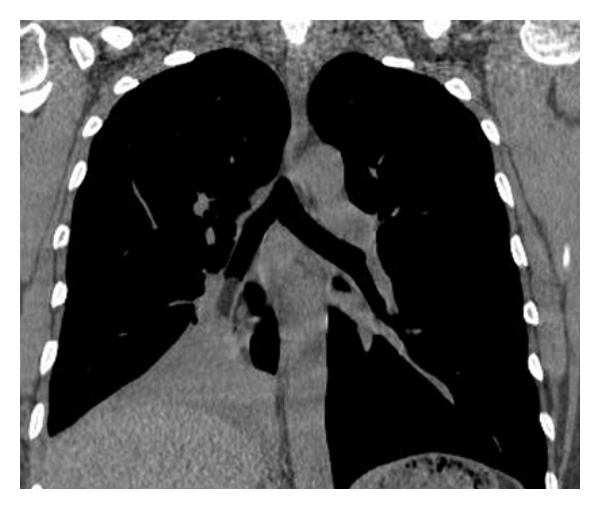
Reformatted reconstruction showed column fatty mass occluding the bronchial lumen.

**Figure 7:**
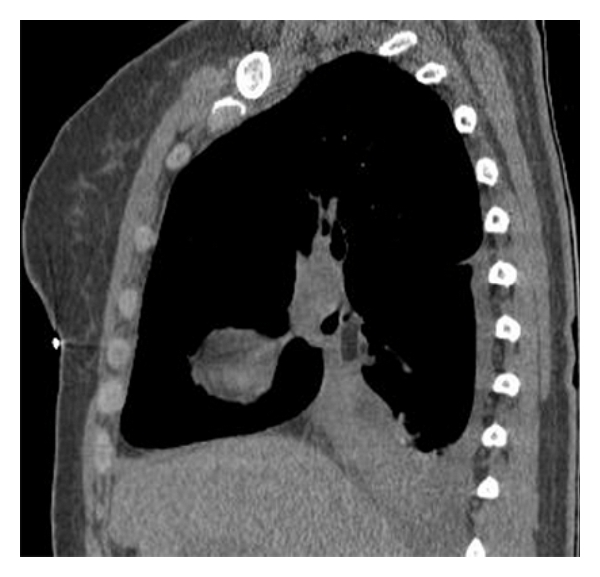
Reformatted reconstruction showed column fatty mass occluding the bronchial lumen.

**Figure 8:**
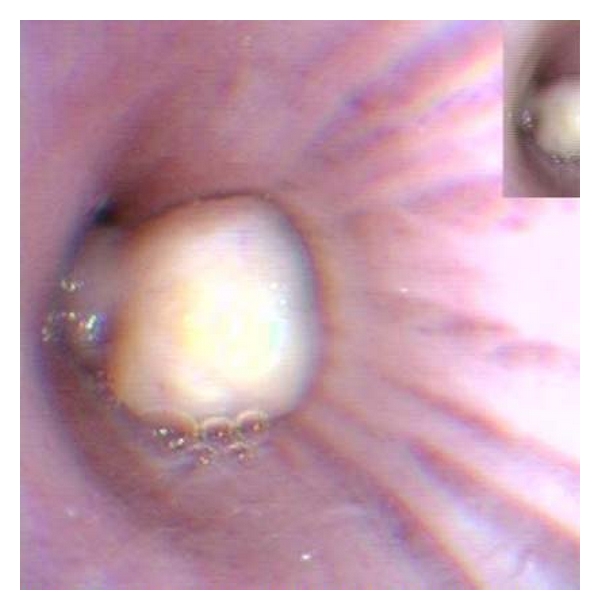
Endoscopy showed smooth yellow mass in the bronchial lumen of right inferior lobe.

A 44-year-old woman was admitted to our hospital complaining of irritating cough for 1 month. All the laboratory values were in the normal range. She had normal chest radiographic findings, so MSCT scan was necessary for further investigation. MSCT revealed a low-attenuation endobronchial mass with CT value of −118 HU and 18 mm × 4 mm in size, obstructing the right inferior basal bronchus. Postobstructive partial atelectasis of the right lower lobe, and a minimal pleural effusion was also found (Figures 5, 6, and 7). Flexible bronchoscopy revealed that a yellow smooth adipose lesion occluded right inferior lobe basal trunk bronchus, and the distal end of the lesion was invisible (Figure 8). The result of biopsy was proved to be fatty tissue. The resection of right inferior lobe was done due to irreversible lung damage. The pathologic examination for endoluminal low-attenuation mass was definitely diagnosed as an endobronchial lipoma, and purulent infection was seen in the distal lung parenchyma. The patient had an uneventful postoperative period and was in good health at the followup of 2 years.

## 3. Discussion

EL is a extremely rare benign tumor and commonly located in the first three subdivisions of the tracheobronchial tree [1, 3, 4]. Most EL originates from submucosal layer of the main or lobular bronchus. The gross appearance of lipomas in the airways is similar to that of lipomas elsewhere, often in the form of soft and circumscribed rounded protuberances of yellow white tissue. Smoking and obesity are significant risk factors for EL in most literatures [4]. However, it remains unclear why exactly heavy smokers have a particularly high incidence of this tumor. Common symptoms of EL include a persistent cough, chest pain, dyspnea, recurrent fever, and pneumonia. Hemoptysis is uncommon, but it can occasionally occur as a result of postobstructive infection. All these clinical features are unable to distinguish EL from other malignant counterparts, such as bronchial carcinoid and mucoepidermoid carcinoma. 

The timely discovery of endobronchial lesions is important to preserve distal lung function before it gets irreversibly destroyed. The signs and symptoms of endobronchial neoplasms cannot be differentiated clinically from those caused by other respiratory diseases. Therefore, imaging studies have a key role in establishing the diagnosis for endobronchial neoplasms. It is often difficult to identify on routine chest radiography, which usually indicates normal or nonspecific postobstructive changes such as atelectasis or pneumonia. Although Matsumura et al. first reported a lipoma identified by conventional CT in 1986, MSCT is the imaging modality of choice for detection and staging of central airway neoplasms so far. MSCT imaging allows for improved multiplanar reformation and three-dimensional reconstruction postprocessing techniques, which complement conventional axial images by providing a more anatomically meaningful display of the neoplasm and its relationship to the adjacent structures, by accurately determining the craniocaudal extent of disease. Simultaneously, MSCT is highly specific and sensitive for endobronchial neoplasms having fatty component like endobronchial lipoma or hamartoma. These imaging information, which can determine amenability of the tumor to surgical resection or other treatment approaches, is crucial for appropriate management planning. Bronchoscopic biopsy is limited sometimes because of the inflamed surface with squamous metaplasia and lack of adequate deep sample, as described in Section 2.1. In such instances, MSCT is an extremely useful diagnostic tool for EL before operation. 

The differential diagnosis of EL is mainly to distinguish it from hamartomas. Endobronchial hamartomas is also a rare benign tumor accounting for 3–20% of all pulmonary hamartomas [5]. Hamartomas may contain cartilage, fat, fibrous tissue, and epithelial components, but endobronchial hamartoma typically contains more fat tissue than parenchymal ones. MSCT can determine the correct diagnosis for fat-containing endobronchial tumor, namely, lipoma or lipomatous hamartoma. Collections of fat alternating with foci of calcification may also be seen in lipomatous hamartoma. When lipomatous hamartoma lacks focal calcification, diagnostic dilemma can arise between them on the basis of MSCT findings. Under this condition, pathological analysis of resected specimen is necessary for accurate diagnosis. However, the distinction of endobronchial lipomatous hamartoma from lipoma is of minor interest as both are rare benign mesenchymal tumors in clinical practice. 

 Although some studies demonstrate that bronchoscopic resection is effective for treatment of EL and suggest that bronchoscopic resection should be considered as the first choice of treatment [4, 6, 7], but under these conditions such as possible complicated malignant tumor, peripheral destructive lung disease due to long-term atelectasis or pneumonia, and expected technical difficulties during procedure, surgical resection has to be considered, as described in our two patients. In conclusion, MSCT is extremely valuable for establishing the diagnosis of EL and also helpful in determining therapeutic planning. 

## References

[1] Raymond GS, Barrie JR (1999). Endobronchial lipoma: helical CT diagnosis. *American Journal of Roentgenology*.

[2] Pollefliet C, Peters K, Janssens A (2009). Endobronchial lipomas: rare benign lung tumors, two case reports. *Journal of Thoracic Oncology*.

[3] Basoglu A, Celik B, Akdag AO, Sengul AT (2004). Endobronchial lipoma: a rare cause of bronchial occlusion. *Interactive Cardiovascular and Thoracic Surgery*.

[4] Muraoka M, Oka T, Akamine S (2003). Endobronchial lipoma: review of 64 cases reported in Japan. *Chest*.

[5] Ahn JM, Im JG, Seo JW (1994). Endobronchial hamartoma: CT findings in three patients. *American Journal of Roentgenology*.

[6] Nassiri AH, Dutau H, Breen D (2008). A multicenter retrospective study investigating the role of interventional bronchoscopic techniques in the management of endobronchial lipomas. *Respiration*.

[7] Choi JC, Yu CM, Ryu YJ (2006). The role of endoscopic surgery for completely obstructive endobronchial benign tumor. *Korean Journal of Internal Medicine*.

